# Barriers, facilitators and motivators of electronic community health information system use among health workers in Ethiopia

**DOI:** 10.3389/fdgth.2023.1162239

**Published:** 2023-06-07

**Authors:** Tariku Nigatu Bogale, Selamawit Meressa Teklehaimanot, Tilahun Fufa Debela, Daniel Berhanie Enyew, Abebe Nigusse Bedada, Segni Dufera Kebebew, Adane Nigusie Weldeab, Dawit Wolde Daka, Herman Jozef Willems, Tadesse Alemu Bekele

**Affiliations:** ^1^ John Snow, Inc. (JSI), Addis Ababa, Ethiopia; ^2^ Department of Health Service Management, School of Public Health, Jimma University, Jimma, Ethiopia; ^3^ Department of Health Informatics, School of Public Health, Haramaya University, Haramaya, Ethiopia; ^4^ Minstry of Health, Addis Ababa, Ethiopia; ^5^ Department of Health Promotion & Health Behavior, Institute of Public Health, University of Gondar, Gondar, Ethiopia; ^6^ John Snow, Inc. (JSI), Boston, MA, United States

**Keywords:** barriers, facilitators, motivators, electronic community health information system, health workers

## Abstract

**Background:**

The electronic community health information system (eCHIS) has been implemented in Ethiopia to support health services delivered by community health workers. Despite the many benefits of digitizing community health information systems, the implementation of the eCHIS is challenged by many barriers resulting in low uptake. This study assessed the barriers, facilitators, and motivators of eCHIS use among health workers with focus on health extension workers (HEWs) in Ethiopia.

**Methods:**

Phenomenological approach was used to assess the barriers, facilitators and motivators of eCHIS use in Amhara, Harari, Oromia, Sidama, South West Ethiopia and Southern Nation Nationalities and People's regions of Ethiopia. Data were collected from 15–29 May 2022. A total of 54 face-to-face in-depth interviews were conducted among HEWs, HEW supervisors, health information technicians and managers. The interviews were audiotaped using Open Data Kit, transcribed verbatim and translated into English. OpenCode 4.03 software was used for coding and categorizing the data. Thematic analysis was used to analyze the data.

**Results:**

The HEWs and other eCHIS users reported lack of infrastructure and resources; poor quality of training, follow-up, and supervision; parallel recording using the manual and electronic system; and HEWs' workload as barriers hindering eCHIS use. Data quality, retrievability, and traceability; tablet portability; encouragement from supervisors; and positive image in the community resulting from HEWs using tablets in their routine activities were the main facilitators of eCHIS use.

**Conclusion:**

The study identified various barriers that adversely affect the use of eCHIS. An integrated and coordinated approach to eCHIS implementation that encompasses removing the barriers, and reinforcing facilitators is required.

## 1. Introduction

The World Health Organization (WHO) recommends training and deploying community health workers (CHWs) in low-and middle-income countries (LMICs) as an effective strategy to address the global shortage of health workers while improving access to, and quality of, primary health care. Training of CHWs, recruited from and deployed to local communities whose culture, beliefs and language they are familiar with, increases access to and utilization of services by target populations. Most CHWs live and work in the community. This helps easily gain the trust of the community and improve the availability and delivery of timely health services. As a result, CHWs have the potential to improve the health and quality of life of rural communities (1).

Ethiopia launched the Health Extension Program (HEP) in 2003. Health Extension Workers (HEWs), the Ethiopian version of CHWs, are female, trained, government employed and salaried frontline health workers that implement the HEP. Close to 40,000 HEWs implement 18 packages of health promotion, disease prevention, and basic curative services at the health post level or by going house to house (2). The HEP enabled Ethiopia to achieve significant improvements in maternal and child health, communicable diseases, hygiene, sanitation, and health care seeking (3).

To capture, track and report data about the implementation of the HEP, Ethiopia started using a manual (paper-based) community health information system (CHIS) in 2008. The CHIS uses a manual family folder, a paper pouch of health information management system which has adopted family as a unit for provision of health services and maintenance of records targeting pregnant women and children for services, and tracking individuals' health. The tickler file system, a box which has slots for the 12 months of the year for filing health cards of clients corresponding to the month of their next visit, helps identify defaulters. The managers at woreda, zonal and regional levels receive regular reports from health posts which they use for planning, monitoring and decision making (4, 5).

Even though the CHIS has shown good report and content completeness, indicators calculation and data display, there are problems in data accuracy and data use at the point of data generation (6). Generally, poor data quality is the main challenge of the health information system in Ethiopia (7, 8). Possible explanations include: using a paper-based system, high cost of printing and distributing formats, tediousness of compiling formats and reports and user errors while recording (9, 10). In addition, the family folder of the CHIS is bulky and vulnerable to damage from rain when carried from house to house. Therefore, many HEWs record in registers instead, transferring the data to the family folders later on which predisposes the system to error and poor data quality (11). Health posts also generate large amounts of data that become manually unmanageable (4).

With the exponential rise of smartphone ownership and associated applications, digital health interventions are being used to support the work of CHWs (12). Community-based information systems (CBIS) and mobile applications are increasingly developed and deployed to quantify and support the services delivered by CHWs (13, 14).

In 2016, Ethiopia identified information revolution (IR) as a major component of its Health Sector Transportation Plan (HSTP-II), a five-year strategic plan document highlighting the roadmap to transform Ethiopia's health sector toward data driven decision making processes and practices. Digitization of the health information system (HIS) and promotion of data use were identified as pillars of the IR transformation. To realize the IR's goals and objectives, the ministry of health (MOH), in collaboration with development partners, developed and deployed an extensive range of digital health tools including the electronic CHIS (eCHIS) (15).

The eCHIS, designed for HEWs, is primarily a mobile-based application that works in an offline environment. The platform allows electronic sharing of household and individual information between HEWs and other staff and helps HEWs to easily monitor updates on patient status. The agrarian eCHIS modules (4 modules composed of HEP packages) are planned to be implemented in 5 releases. The first two releases (release 1 and 2) (composed of household registration, reproductive, maternal and child health (RMNCH), disease prevention and control (DPC), and logistics and supplies management) have been implemented in more than 7,000 health posts (HPs). Release 3–5 composed of the remaining packages of the HEP, are currently in the pilot phase (16).

The eCHIS has many benefits. It is a convenient way to save lives and improve care, particularly in low-resourced community settings (17). Data accuracy, integrity, and decision-making at the community level tend to be better when using eCHIS than paper-based CHIS (18). Additional benefits include electronic decision support (19); faster referrals; patient tracking; minimization of duplication in recording and reporting; and enhancement of communication between providers (20, 21).

The eCHIS is being scaled up in phases due to the need for high upfront investment for infrastructure and other costs attached to it. The belief is that the long term return on investment (ROI) including its expected outputs are better compared with the manual system. This is also in line with Ethiopia's plan of going digital by 2025. Implementing eCHIS is not a panacea for all health care challenges in Ethiopia. But it is believed to address part of the health care challenges of communities in the country (22).

Despite the many benefits of digitizing health information systems, studies show that the implementation is constrained by many barriers that include: health worker education level (23); resistance of the health workforce to use digital tools (24); unreliability or absence of infrastructure (e.g., electricity and network) (23, 25); interoperability problems (20, 26); lack of coordinated actions among the different actors involved in digital health information system (24); lack of political support and user acceptance (27); privacy and confidentiality concerns (26, 28); limited access to cell phones (25); economic and legal factors (24, 29); cell phone theft and security; absence of local skills in programming and technological operation (23); lack of trust among stakeholders, particularly making disparate pre-existing systems, owned by different stakeholders (26, 27); and inability to procure and use systems that comply with national data and interoperability standards (21).

The eCHIS requires reliable connectivity to send and receive data and electric power to run the application. However, access to connectivity and electric power is limited in sub-Saharan Africa including Ethiopia. Ethiopian telecommunication services, the only telecom services operator owned by the government with recent signs of opening to the private sector, provides around 85% of Ethiopians with at least 2G mobile coverage, 66% with 3G but just only 4% with 4G connectivity. In rural areas where HEWs live and work, connections are usually intermittent or absent. Ethiopia also has a large divide in electricity access between urban and rural settings. Most large urban areas have nearly universal electricity coverage. In large towns, 95% of people have electricity; though blackouts are common, compared to 83% of people in small towns. However, the situation differs markedly in rural areas. According to the Living Standards Measurement Study, only 9% of people living in rural areas (i.e., excluding small towns) have access to electricity (30). To address this challenge, the MOH procured and distributed 29,950 power banks, charging devices that store electric energy, for off-grid settings together with tablets (31). The level of training of CHWs is another challenge affecting their level of comfort with the technology (13). An evaluation of a community-based health information system in Kenya highlights the need for intensive training with periodic refresher courses for CHWs involved in data collection (32). WHO and Community Health Impact Coalition (CHIC) also advocate for increased and sustained financial investment to remove barriers to CHWs and to strengthen and expand national CHW programs (1, 33).

To promote a rapid adoption of digital technologies including the eCHIS, Ethiopia has been training and deploying Health Information Technicians (HITs). The HITs are trained for three years on electronic health information systems and they support health workers including HEWs to (a) improve their computer skills, (b) report health data upwards in the system and, (c) extract health data for local use to improve the quality of care (3).

The success of digital health technologies strongly depends on the uptake and appropriate use by healthcare professionals (24). This requires continuously identifying and addressing barriers and strengthening facilitators. This study assessed barriers, facilitators and motivators of eCHIS use among health workers with focus on HEWs in Ethiopia.

## 2. Materials and methods

### 2.1. Study design, setting and period

The study used a phenomenological approach. This approach is used to identify the essence of human experiences about a phenomenon as described by participants (34). We used the approach to explore the lived experiences of HEWs in using eCHIS in their routine work. Data were collected from six regions of Ethiopia (Amhara, Harari, Oromia, Sidama, Southern Nation Nationalities and People's, South West Ethiopia) from 15–29 May 2022.

### 2.2. Study population and eligibility criteria

Data were collected from HEWs, HEW supervisors, health information technicians (HITs), and woreda health officials (health managers at district health offices). Health workers who were willing, 18 years or older and who provided written informed consent participated the study.

### 2.3. Sample size

Reaching a point of saturation is usually considered sufficient instead of pre-determining a sample size (35). However, most studies suggest 5–50 interviews as adequate (36). In this study, 30 in-depth interviews (IDIs) were conducted with HEWs, 24 IDIs (8 interviews each) were conducted among HITs, HEW supervisors and woreda health officials. The IDIs were distributed proportionally across regions where the study was conducted.

### 2.4. Sampling technique and procedures

A purposive sampling technique was used to select HEWs, HEW supervisors, HITs, and woreda health officials. The respondents were selected in health posts or woredas where eCHIS has been implemented for at least 6 months. Only one HEW (the one with the most eCHIS experience) was selected and interviewed from each health post.

### 2.5. Data collection tools and procedures

An in-depth interview (IDI) guide was developed, reviewed by experts, and used in the study. The English version of the IDI was translated into Amharic and Afan Oromo languages and pretested before data collection. The guide included questions that address the barriers, facilitators and motivators of eCHIS use. Data collection was carried out by trained and experienced qualitative data collectors. The data collectors were trained for two days and data were collected using a GPS enabled Open Data Kit (ODK). The interviews were tape recorded and supplemental notes were taken during the interview. Probing questions were used to explore issues in depth.

### 2.6. Data analysis

The audio records of the interviews were transcribed verbatim in the language of interview and then translated into English. The translated data were cross-checked with the audio file to ensure proper transcription and translation. The data were migrated to OpenCode 4.03 software for coding and analysis. Hybrid coding was used to reduce the data and similar codes were combined to produce categories. Thematic analysis was used for data analysis.

### 2.7. Trustworthiness and eliminating sources of bias

Trustworthiness of this study was ensured through: peer scrutiny of the research (e.g., when ideas were not clear, either the respondent or person who knew the subject were contacted for insights and meanings); in-depth methodological descriptions and by ensuring information saturation. A systematic review conducted on the influence of political context in public health research in Ethiopia showed the possibility of social desirability bias during reporting the benefits of the HEP (37, 38). However, data collection for this study was done by experienced and trained persons and the confidentiality of the data was clearly communicated to the respondents to minimize such biases.

### 2.8. Ethical considerations

Ethical clearance was obtained from the Institutional Review Board of the Ethiopian Public Health Institute (Approval number EPHI-IRB-389-2021 dated 8/10/2021). Then, permission was obtained from local administrations in the study areas. During data collection, written informed consent was taken by the data collectors in a setting that allowed privacy. Respondents were informed about their voluntary participation in the study. In addition, they were told about the potential benefits, harms, confidentiality and the possibility of withdrawing from the interview without giving any reasons. Confidentiality of the information was maintained, and the data were recorded anonymously throughout the study. The transcripts were given different codes for each respondent to maintain the confidentiality of the information relating to each participant. The recordings and transcripts were stored in a password protected computer. Notes and any other recordings were destroyed once the summary was prepared.

## 3. Results

### 3.1. Socio-demographic characteristics of health workers

A total of 54 IDIs were conducted with HEWs, HEW supervisors, HITs and Woreda health officials in the six regions across Ethiopia. The mean (±SD) age of the participants was 30.2 (±5.5) years. Most of them (90.7%) were married, and 31 (57.4%) lived in urban areas. Twenty-eight (51.9%) of the HEWs were trained at Level IV which is a diploma level certification of HEWs, with median (±IQR) experience of 11.0 (±7.3) years. Most (87.0%) of the participants received training on eCHIS (Table 1).

**Table 1:** Socio-demographic characteristics of health workers, Ethiopia, June 2022 (*n* = 54).

Variable	Category	Frequency	Percent
Age (in years)	20–30	30	55.6
	31–41	24	44.4
Marital status	Married	49	90.7
	Single	5	9.3
Residence	Rural	23	42.6
	Urban	31	57.4
Education	Level III	10	18.5
	Level IV	28	51.9
	Degree	16	29.6
Professional category	HEWs	30	55.6
	HITs	8	14.8
	HEW supervisor	8	14.8
	Woreda health official	8	14.8
Experience (in years)	<5	9	16.7
	5–10	17	31.5
	>10	28	51.9
Received training on eCHIS	Yes	47	87.0
	No	7	13.0
eCHIS training by professional category	HEWs	27	57.5
	HITs	7	14.9
	HEW supervisor	8	17.0
	WorHOs	5	10.6

### 3.2. Barriers against use of eCHIS

The qualitative study identified multiple barriers against use of eCHIS operating at many levels. Generally, six themes emerged explaining eCHIS use barriers: policy, infrastructure, training, human and system, management and administration, and resources.

#### 3.2.1. Policy implementation

Current MOH's direction demands parallel implementation of the manual CHIS and eCHIS. This is causing a significant data collection burden among HEWs. Almost all HEWs reported the burden of parallel recording using the manual CHIS and the eCHIS as a major challenge. Most of the HEWs reported that they usually transfer data from the manual family folder to the eCHIS after service delivery. This has become a source of additional workload, fatigue and dissatisfaction which can negatively affect subsequent scale up efforts. The fact that HEWs are not using eCHIS during actual service delivery affects the service and data quality, the access to real time data, and the virtual monitoring of HEWs performance.

*First, I record households' information on paper, and then record it on eCHIS at home after I return from work. (HEW from Deder, Harari Region)*

Even if HEWs are now allowed to fully switch to eCHIS for the first two releases that are already digitized, it is mandatory for them to always carry and use the manual CHIS as well to register services on HEP program components that are not yet digitized (the remaining three releases). Some thought will need to be given to whether phasing the release of the modules is the best strategy or if it would be better to implement all the releases at the same time. The latter may be more appropriate to limit HEW workload and fatigue due to parallel recording. In addition, it would allow the MOH to evaluate the full picture of the eCHIS implementation, document lessons including the costing and use the learning to guide the scale up.

*Simultaneous use of paper and eCHIS is a factor affecting HEWs use of eCHIS negatively. HEWs complain that they are overburdened by using both paper and eCHIS at the same time. (HIT from Sire Woreda, Oromia Region)*

Each HEW is given a tablet for eCHIS implementation. Most HEWs have their own phones. To reduce the burden of carrying two phones and avoid robbery or theft, HEWs usually carry the eCHIS tablet only and hence use it for their personal issues (dialing, receiving calls, downloading different applications including videos, taking pictures and videos). This may lead to reduced storage capacity and tablet processing speed. HEWs, on the other hand, also use their own phones for HEP related activities such as calling ambulances for referral purposes. Therefore, there is a need to balance between use of eCHIS tablets for personal business and work.

*It is difficult for them (HEWs) to carry both the tablet and phone during home to home visit. They are afraid of being exposed to theft. Some even use their tablet for personal purpose. (HIT from Sokoru Woreda, Oromia Region)*

#### 3.2.2. Infrastructure

Most HEWs reported that infrastructure such as lack of properly functioning tablets, SIM cards, connectivity, server and power supply affect their use of eCHIS.

##### 3.2.2.1. Connectivity (SIM card and airtime)

Almost all HEWs mentioned lack of consistent refilling of airtime, pre or postpaid top-up to access network for internet, voice call or short message services, which affect their ability to send and receive referral data, sync health service delivery and household (HH) profile registration information to server, back-up their data and receive version updates. HEWs sometimes use their own funds to recharge airtime for eCHIS related activities.

*I sync data using my own mobile data. Sometimes, higher officials at the woreda level are not recharging airtime timely.* (A HEW from Wuchale Woreda, Oromia Region).

In some HPs, HEWs were not given SIM cards. In other places, HEWs reported loss and non-functionality of SIM cards.

*Not all of the SIM cards given to us were functional. It can't send data to the server. One year back, about 110 households' data were lost from the tablet because of lack of SIM card* (HEW from Wuchale Woreda, Oromia Region)

Some HEWs had to travel to woreda health offices, hotels or Wi-Fi hotspot areas to get connectivity to sync their data. This causes service interruptions in some health posts. It also incurs travel cost to the HEWs. In some places, HEWs reported that mobile network strength is weak and syncing time is long. This discourages HEWs from using eCHIS.

*I have to go to the nearby hotels or other areas where there is Wi-Fi to sync data, or you have to wait overnight for the mobile data/network to work to sync the data.* (HEW from Sodo Woreda, SNNPR)

*There is a network problem in our Woreda. In some Kebeles, there is no network at all. In addition, SIM cards are not functional and hence HEWs can't sync data to the server.* (Woreda health official from Wuchale Woreda, Oromia Region).

##### 3.2.2.2. Hardware (tablets and server)

Almost all HEWs included in the study had tablets for eCHIS. However, most of them reported slow processing capacity of tablets. As a result, the use of eCHIS is delaying the accomplishment of HH registration and service delivery leading HEWs to resort to the manual (paper based) system (CHIS).

*While clients are with us, we (HEWs) spend long time without doing one. Therefore, we write it (the service data) on a piece of paper and transfer it to the tablet later.* (HEW from West Badawacho in SNNPR)

The capacity of the central server determines the syncing time. HEWs reported that the central server at MOH has limited capacity which prolongs the time to sync data.

*Server is too slow. There was an instance when full data of one Woreda was lost from the server as it took time to sync.* (HEW Supervisor from Jabitehnan Woreda, Amhara Region).

##### 3.2.2.3. Power supply related barriers

Access to electricity in rural areas, where most of the HPs are located, is very limited. In some places, eCHIS users reported lack of power banks and frequent interruption of electricity as a hindrance to eCHIS use. This has been a major challenge particularly in remote areas where the reach of the national electric grid is limited.

*Those, in remote areas, who don’t have access to electricity or have no power banks have to wait (to use eCHIS) until the tablet is charged at the nearby town or health center.* (HEW supervisor from Sofi Woreda, Harari Region).

But even HEWs, who have power banks, reported frequent depletion of power reserves because of low power storage capacity. As a result, they have to travel hours to days to a nearby town to charge their power bank. This results in service interruption, leading to additional workload and discouragement to use eCHIS. This was even possible for HEWs working close to towns.

*The charge runs out quickly. They (HEWs) send their tablet to Deder town for recharging which may take two days.* (HEW supervisor from Deder Woreda, Harari Region).

In rare cases, where the kebele has rugged terrain, conducting house to house visits requires HEWs to spend nights in the villages or “*Gots*” before returning to their base.

*Those who don’t have power banks and access to electricity in remote areas have to wait until it is charged to use eCHIS.* (HEW Supervisor from Sofi Woreda, Harari Region).

Some HEWs reported lack of a readily available and timely maintenance and troubleshooting as a barrier for use of eCHIS.

*This past two weeks, the tablet available in the health post malfunctioned and it was sent for maintenance. Because of this problem, it was not possible to use eCHIS.* (HEW from Wuchale Woreda, Oromia Region)

#### 3.2.3. Training related barriers

The digitization of the agrarian Health Extension Program is being implemented in 5 separate releases. As a result, the HEWs training on eCHIS is being delivered release by release in a phased manner.

##### 3.2.3.1. Lack of training quality

The eCHIS training is designed to be delivered through theoretical, practical and off-site demonstration sessions followed by post-training on-site supportive supervision, mentoring, coaching, and feedback. Following the basic training, the HITs and HEW supervisors are responsible to provide on the job training to HEWs which helps to initiate and maintain the continuity of eCHIS including providing on the job training to replace staff leaving through attrition, transfer or new assignments. Similarly, HEW supervisors provide orientation to newly hired HEWs on eCHIS before a formal training is organized.

There are varying views regarding the sufficiency of the eCHIS training to HEWs. Most HEWs mentioned that the duration and quality of the training is insufficient to impart the necessary knowledge and skill needed to use eCHIS in their routine activities.

*I don't believe I have acquired the necessary knowledge and skill during those four days of training.* (HEW from Jabitehnan Woreda, Amhara Region)

The duration of the training for the first release of the eCHIS is reported to have been inconsistent varying from 4 to 6 days, which in some training was at the expense of skipping content to shorten the duration. The national standard for the training of the first release of the module is 6 days.

*I didn't acquire basic knowledge and skill in those areas from the training I received. The training was shallow and the trainer skipped pertinent information.* (HEW from West Badawacho Woread, SNNPR)

In addition, absence of sufficient demonstration and practical sessions during training was widely mentioned by HEWs. In some places the training was merely theoretical, leaving HEWs unable to use it during their regular work.

*We did not have sufficient demonstration sessions during the training. This has created a skill gap and impeded our ability to properly operate the technology*. (HEW from Deder Woreda, Harari Region)

Most HEWs expressed a lack of confidence and competence in use of the eCHIS application due to their lack of knowledge. Most eCHIS users, particularly HEWs, associate it with the poor quality of eCHIS training and the absence of regular post-training follow up.

*We (HEWs) forget it (eCHIS) over time, especially if you do not implement it properly after the training. It needs frequent practice.* (HEW from Sofi Woreda, Harari Region)

Immediate planning of post-training follow-up helps to provide tailored support to trainees, understand and solve contextual challenges and ensure initiation of services before important skills and knowledge are lost. However, almost all HEWs raised the lack of post-training follow-up as a barrier to the use of eCHIS. Some woreda health offices reported that there was insufficient budget for post-training follow-up and supportive supervision.

*We (HEWs) were trained in 2021 but we started using the application for household registration and service delivery this year* (2022)*.* (HEW from Deder Woreda, Harari Region)

Most of the time, eCHIS training for HEWs is organized and delivered in the same classroom with a highly diverse mix of professionals including HITs, HEW supervisors, midwives and woreda health office. Hence, the training is not tailored to HEWs’ specific needs considering their educational background and their limited prior exposure to technology. Since HITs are IT professionals, it is easier for them to understand the eCHIS training content.

*There is also a problem of not properly training the HEWs. We are trained together in the same session. I am a Health Informatics Professional and there is nothing difficult for me. It is better to train them separately.* (HIT from Fagita Lekoma Woreda, Amhara Region).

##### 3.2.3.2. Lack of training

Primary health care unit (PHCU) directors and woreda health office heads, who are supposed to supervise and monitor eCHIS implementation activities, are not trained on eCHIS. This has resulted in their inability to coordinate and support the eCHIS implementation and discharge their supervisory responsibilities.

*The head of our Woreda health office has not received training. So, how can he monitor and review this work? Many supervisors from the health center have not received the training either. When they go to the health post for supervision, they can't support the HEWs as intended.* (HEW supervisor from West Badawacho Woreda, SNNPR)

In a few places, HEWs mentioned that they did not receive any training except orientation from colleagues or supervisors on eCHIS despite expectations to use the application in their routine work. Common reasons for not being trained include: absence due to maternity leave, formal education and assignments in other kebeles while eCHIS training was taking place in their current location, and new hires.

*I haven't attended the training. I was on maternity leave but I was given orientation.* (HEW from Sokoru Woreda, Oromia Region)

##### 3.2.3.3. Lack of consistent training approach and directions

The implementation priority of the modules under the first release was inconsistent across different regions and woredas. Despite some HEWs receiving training on HH registration and service delivery at the same time, local officials prioritize the completion of HH registration before using eCHIS for service delivery. In some woredas, HEWs mentioned that the completion of HH registration is mandatory before using eCHIS for service delivery. Hence, skills on use of the eCHIS for service delivery is lost until HEWs complete the HH registration. In most places, HEWs are required to transition to the service delivery application without refresher training.

*It has been a while since I received the training (eCHIS). I will be providing services using the app soon once we (HEWs) complete registering the households. Unless I am given refresher training, it will be very difficult for me to provide services using the system.* (HEW from Sofi Woreda, Harari Region)

In some places, HEWs are not getting technical support including referral feedback from health centers due to lack of the necessary skills and knowledge on eCHIS among staff at health centers. In some places, health center level staff are not practicing eCHIS as intended, resulting in important loss of eCHIS skills over time.

*Three individuals received training on eCHIS. They know eCHIS in theory. But you (meant to generalize the fact) forget if you do not practice it.* (HEW from Wuchale Woreda, Oromia Region)

#### 3.2.4. Human and system factors

This refers to the challenges related to user friendliness, simplicity, attachment, exposure and adaptability to the eCHIS application.

Some of the HEWs mentioned that navigating through the different pages of the eCHIS has been a challenge during service delivery. Challenges reported include being stuck on a page and involuntarily restarting of the eCHIS application without saving the data previously entered. This has resulted in loss of data and discouraged HEWs from using the application.

*It (the eCHIS) sometimes erases what you did and takes you back where you started. Then you have to do it again.* (HEW from Deder Wored, Harari Region)

*Sometimes we (HEWs) lose data during the data syncing process as the application formats itself on its own.* (HEW from Sodo Woreda, SNNPR)

Some HEWs mentioned they have suffered from eye problems including blurred vision and itching as they were using the tablets continuously (day and night) to complete HH registration.

*In the beginning, we (HEWs) use the tablets continually for long hours to finish the household head and family members' registration. As a result, we suffered with eye problems like burning and itching.* (HIT from Sodo Woreda, SNNPR)

Prior exposure to smartphones and other technologies among HEWs is important for proper utilization of eCHIS. Almost all HEWs in this study mentioned that they have no experience using touch screen smartphones.

*Most of the health extension workers did not have touch screen mobile phones before the introduction of eCHIS.* (A HEW supervisor Sodo Woreda, SNNPR)

While the eCHIS provides opportunity for HEWs to get to use smartphones, the inadequacy and quality of the training including the post-training follow-up support is limiting their ability to interact with eCHIS.

*Most of us are new to this kind of technology. Thus, it is difficult for us to accept and use the technology easily* (HEW from Deder Woreda, Harari Region).

The risk of losing data when a tablet is stolen, especially if it is not synchronized and the subsequent breach of confidentiality should be considered while using digital technologies for health services delivery. Most of the HEWs stated that they are afraid of theft and robbery during home-to-home visits for registration and service delivery. Hence, they refrain from using the tablet during HH visits to avoid the risk.

*There is a HEW who was exposed to theft and held accountable to pay about 8,000 ETB (Ethiopian currency name). So, there is a fear.* (HEW from Wuchale Woreda, Oromia Region)

#### 3.2.5. Management and administrative

Management and administrative barriers refer to issues related to planning, organizing, directing, controlling (monitoring, supervision coaching, and feedback), communication, budgeting and staffing.

Generally, the national health management information system (HMIS) has principles of simplification, integration, standardization, and institutionalization of the HMIS implementation. Simplification includes reducing the data collection burden of health workers at all levels, including HEWs. In this regard, the assistance of technology, such as eCHIS, is critical. However, most HEWs reported that the data volume of HH registrations is enormous and that the data collection forms are too complex, consuming a significant amount of their time. This is on top of the time taken to travel from base locations to homes and from home to home.

*It is better for me to write it down on paper. When I register everything directly on the tablet, it will take me longer; that's why I do not use it.* (HEW from West Badawacho Woreda, SNNPR)

To initiate use of eCHIS, MOH requires HEWs first update the information on the manual CHIS. Then, move the information from the manual CHIS to the eCHIS later. Most HEWs reported this as a time consuming and cumbersome process discouraging eCHIS use. This direction has led HEWs to develop fatigue.

*Continuously updating the data (on the manual CHIS) has prevented us from using eCHIS. It (updating of the manual folder) has taken us a long time. Until we finish updating the e-CHIS application, we can’t do anything.* (HEW from Fagita Lekoma Woreda, Amhara Region)

Generally, HEWs mentioned that close supportive supervision encourages them to use eCHIS for HH registration and service delivery.

*Encouragement, feedback and follow-up from supervisors increases my commitment to implement eCHIS as per the intended plan.* (HEW from Sire Woreda, Oromia Region)

Supervision helps ensure delivery of materials (for example, SIM cards, power banks, manuals etc.), monitor the status of HH registration activities, conduct HEW performance evaluation and provide feedback, ensure initiation of eCHIS based service delivery, support data use for decision making, and provide troubleshooting support for tablets for sustainable eCHIS implementation. However, almost all HEWs interviewed reported lack of regular supervision from woreda health offices and health centers. This has led to a highly pronounced skill gap in eCHIS implementation.

*Lack of supportive supervision and commitment is a problem. Strengthening supportive supervision is compulsory to improve the utilization of eCHIS.* (Woreda Health official from Sokoru Woreda, Oromia Region)

Almost all woreda health offices and health centers reported lack of adequate budget for conducting regular supportive supervision and review meetings hampering their ability to monitor the status of eCHIS implementation, identify needs and build HEWs capacity, and facilitate the sharing of experiences among HEWs.

*In our woreda, there is no budget for supportive supervision. We (the woreda health office) can't give any per diem when they visit health posts. We have no budget for monitoring and evaluation. We conduct a review meeting without providing any per diem or any payment for transportation.* (Woreda health office head from Wuchale Woreda, Oromia Region)

Effective implementation of eCHIS requires full commitment at all levels. Most HEWs reported poor commitment and leadership at Woreda level affecting their proper use of eCHIS for HH registration and service delivery. However, HEWs' commitment and willingness to use eCHIS have been reported to be very important as well.

*There is lack of leadership commitment. This is because they pardon those workers whom we punish or warn when they do not implement properly, this may affect the work.* (HIT from Sofi Woreda, Harari Region)

#### 3.2.6. Resources

The implementation of eCHIS requires financial, time, human resource, space, infrastructure and other material resources. The MOH and its partners have mobilized considerable resources for its effective implementation. However, challenges with the quality, quantity and type of resources needed to implement eCHIS persist. Some health posts are staffed below the national standard. This increases the workload on the available HEWs.

*We (the woreda) haven’t finished household registration because 15 HEWs left for education and they returned the tablet to the store. So, 15 tablets are out of work at this time.* (Woreda health office head from Wuchale Woreda, Oromia Region)

Almost all HEWs mentioned lack of adequate human resources in light of the burden of competing priorities, such as their required participation in frequent campaigns [e.g., immunization, mass deworming and vitamin A supplementation, community-based health insurance implementation (CBHI), open defecation prevention activities, emergency response activities such as COVID-19, trachoma mass treatment]. This regularly diverts their attention from doing their regular community-based work. While involvement in campaigns is considered one of the responsibilities of HEWs, frequent engagements stretch them thin and prevent them from focusing on HH registration and service delivery.

*At a time when eCHIS is in full implementation, campaigns are on the rise. And we have a hard time implementing it (eCHIS) here. Currently, we are working on seven campaigns*. (HEW from Jabitehnan, Amhara Region)

In few places, HEWs left their workplaces for education and maternity leave, aggravating the human resource needs for eCHIS implementation.

*There are only two HEWs in one health post but the population is large. There are 8,000 households in one kebele and we can't address it even in a year. If possible, it would be good if they can provide us with additional human resources; it is difficult for two HEWs to serve 8,000 households.* (HEW from Oromia region, Deder Woreda)

### 3.3. Facilitators of eCHIS use

The study also explored the facilitators of eCHIS use. Accordingly, tablet portability, data retrievability, transparency and traceability, and data quality were identified as major facilitators of use.

#### 3.3.1. Tablet portability

Nowadays, using technology like tablets in community health service is increasingly common for household registration, service delivery and accessing patient information. The smaller the size of the tablet, the more comfortable and safer it is for HEWs when they travel from house to house. Most HEWs included in this study reported that the size of the tablet is easy to carry when they travel from their base locations to villages during home visit.

*We carry the tablet in our hands and it is simple.* (HEW from Jabitehnan Woreda, Amhara Region)

Furthermore, most respondents mentioned that the portability of tablets allowed them to readily access data wherever and whenever they needed: be it in the villages during home visit, at the health posts or at their homes, and some of them described the tablets as more comfortable than the manual CHIS.

*The tablet allowed us to access data that we (the woreda staff) want at any time. The tablet is also portable and requires no space unlike the paper-based folder method of recording.* (Woreda health office head from Jabitehnan, Amhara Region)

#### 3.3.2. Retrievability

The way individuals' health information is stored affects accessibility of data, a key concern for health workers. Most respondents mentioned that the manual CHIS was challenging for them because data stored on the hardcopy can be lost or damaged by fire or rain. They reported that eCHIS saves data on a server and it has made it possible for them to easily access data when a tablet is lost. They also mentioned that eCHIS saves time and simplifies work compared to the manual CHIS.

*The paper-based work was challenging for us. eCHIS saves data on a server and you can easily access your data even if you lose your tablet phone as long as you have the password and username.* (HIT from Sofi Woreda, Harari stated)

#### 3.3.3. Traceability and transparency

Some HITs mentioned that HEWs use of eCHIS is helping them track house to house service delivery by tracking HEWs through the GPS system installed on the eCHIS.

*I monitor whether or not they are actually going from house to house for household registration. It is possible to monitor the HEWs because this system has GPS. I can access their data… I can use it to trace them.* (HIT from Sofi Woreda, Harari Region)

Both HITs and woreda health officials use eCHIS to remotely monitor the day-to-day activities of HEWs. This creates accountability, increases effectiveness, and ensures the availability of HEP services.

*I use these to cross-check whether everyone in the catchment area has worked properly or not. I especially use these things to focus on identifying what works and what doesn’t.* (HIT from Jabitehnan Woreda, Amhara Region)

Woreda and health center level visibility of HEW performance helps to monitor HEP implementation using the eCHIS dashboards. The dashboard is used to conduct performance review meetings, usually comparing plan vs. performance.

*Based on their performances, we have identified the lower performing HPs and conducted a review meeting with them separately for improvement of the utilization of eCHIS* (HEW supervisor at Sokoru Woreda, Oromia Region)

#### 3.3.4. Data and services quality

Most respondents reported that the use of eCHIS for HH registration and service delivery has greatly improved the accuracy, completeness and credibility of data compared to the manual CHIS, which encourages data use.

*The eCHIS improved data completeness, avoided the recording of false data, avoided data duplication, saved time and resources, and improved respect by the communities*. (HIT from Jabitehnan, Amhara Region)

### 3.4. Motivations to use eCHIS

This study found encouragement and image as important motivational factors for eCHIS use. Almost all HEWs included in the study mentioned that they were encouraged to use eCHIS for its various benefits. The fact that eCHIS saves time to do HEP activities is encouraging the HEWs to use the system.

*Using eCHIS has many benefits. For instance, it saves our time to give services. This is encouraging us to use eCHIS.* (HEW from Sire Woreda, Oromia Region said)

In addition, simplification and user-friendliness are mentioned by some HEWs as an encouragement to use eCHIS. eCHIS is also helping HITs and HEW supervisors to remotely access data and monitor HEWs performance which they said motivates them to use eCHIS.

*It is easy to know whether or not a given HEW recorded data sitting at her home. This is because they (HEWs) capture GPS data. This is encouraging.* (Woreda health official from Sofi Woreda in Harari Region)

The application is facilitating proper recognition and encouragement by avoiding false reports. This encourages honest and hardworking HEWs to get recognized based on quality assured data. Before the implementation of eCHIS, performance-based awards and recognition to HEWs was given based on data of which the quality was not verified. With eCHIS, supervisors and woreda health officials can verify actual home visits as eCHIS enabled the capturing of geo-location information. Some HEWs also reported that the eCHIS application has improved the credibility of reports they generate particularly regarding community activities (because of geo-location information). This has been mentioned as a motivation to use the eCHIS application by most respondents.

*It is also possible to differentiate the best from the poor performers. These are some of the points which encourage me to use eCHIS in my work. (*HEW from Sire, Oromia Region)

*You cannot lie. It asks for the GPS of that specific family. Thus, it is impossible to report false data as everything about that household needs to be registered.* (HEW from Sofi, Harari Region)

Supportive supervision creates a more supportive, caring and positive work environment as it provides a space for regular communication, problem solving, enhanced accountability, increased sense of being supported, development of professional skills, and increased team work. Some HEWs reported that supervision and feedback encouraged them to continue using eCHIS in their work.

*Supportive supervision from the woreda health office and health center motivate me to use eCHIS.* (HEW from Sire, Oromia Region)

Almost all HEWs live and work in rural areas where access to technology is limited. The opportunity for exposure to current ICT has been reported as a motivation for most HEWs and HITs to use eCHIS.

*What inspires me is the technology, and I enjoy it and I want to use electronics and different apps. It is something new. So, I am eager to learn something new*. (HIT from West Badawacho, SNNPR)

#### 3.4.1. Image

Image is the degree to which use of a technology is perceived to enhance one's status in one's social system. HEWs don't live in isolation, they live in communities, with colleague HEWs, HEW supervisors, HITs and woreda health office staff. Support from this social system is important for HEWs to use eCHIS in their routine activities.

*Support from health center focal, woreda health office head and my co-workers encourage me to use the app.* (HEW from Sodo, SNNPR)

Recognition and respect are important motivation factors for people to adopt certain behaviors. Most HEWs and HEW supervisors included in the study reported earning more respect and recognition from communities within which they live and work, as a result of using tablets during HH registration and service delivery.

*Communities respect us while we provide services using tablets. This increased our acceptability at community level and enabled us to use eCHIS in our routine work.* (HEW from Sire, Oromia Region)

## 4. Discussion

This study explored the barriers, facilitators and motivators of eCHIS use among health workers in Ethiopia. Health workers included in this study reported a number of interrelated barriers categorized into six themes hindering eCHIS utilization. They also described some of the benefits, motivators and facilitators of using the application in their routine work.

Limited connectivity due to poor network coverage, inconsistent air-time recharging and non-functionality of SIM cards were among the main barriers related to infrastructure. Some of the health workers used their own funding to establish connectivity. These findings are in accordance with barriers that were identified and reported in other African, Asian and South American countries (16, 39–44). Power shortage and frequent interruption of electricity to charge tablets were reported as barriers hindering the utilization of eCHIS. Different studies conducted across many settings also reported power shortage and lack of electricity to use digital platforms (39, 42, 45–47). Low processing speed and storage capacity of tablets, and lack of readily available tablet maintenance services were major challenges reported by many eCHIS users. Studies in Brazil, UK and Rwanda showed that storage capacity and processing speed are important parameters that affect use of digital community health information systems (48–50). This may create extra burden, frustration and loss of hope among health workers discouraging consistent use of the application. Furthermore, this may drive health workers to switch back to the paper-based system.

Health workers included in the present study felt that the lack of integration challenged them to easily access patient information, analyze data and generate reports. This added another layer of complexity by increasing workload among the healthcare professionals. This may also reduce their level of motivation further restricting the utilization of eCHIS. A study reported that high workload and low pay are important demotivating factors for HEWs performance (51). Other studies reported that system integration facilitated health workers' access to client information across different care points within the health system, and stimulated health workers to use technology in their routine work (34, 50).

In general, the success of eCHIS implementation depends on the presence of a well-established information technology infrastructure. Prioritizing and investing in eCHIS infrastructure will create an environment that will provide reliable internet connectivity, power supply and availability of reliable devices with readily available maintenance services which will ensure subsequent successful eCHIS implementation and use. Moreover, it is important to ensure the resilience of eCHIS infrastructure that withstands natural or man-made disasters. For example, the recent conflict in northern part of Ethiopia has led not only to infrastructure damage but also to loss of patient and client information. Hence, investment in infrastructure requires current and anticipated local contexts into consideration.

Poor quality training creates a feeling of insecurity among health workers, reducing their interest in using technology. This will lead to errors while using the technology affecting the quality of care and data. In this study, inadequate training, short training duration, and the lack of practical sessions, refresher training, follow-up and supervision were reported as barriers hindering the use of eCHIS. Various studies identified that inadequate training, lack of refresher training, and absence of technical support and follow-up made health workers feel insecure and less inclined to use technologies (44, 49, 52–54).

Technology literacy, user-friendliness and simplicity of applications are important for successful use of digital tools in the healthcare system. In this study, limited prior exposure to technology and lack of smartphone ownership were challenges to use eCHIS. Similarly, many studies reported that poor digital literacy (unfamiliarity) and lack of user friendliness of applications frustrated health workers and caused reluctance to use technology for service delivery (43–45, 48, 52, 54–56). Damage, loss and theft (because health workers have to carry both personal and work phones) and the fear of them were additional bottlenecks health workers faced. Similar findings were reported in other studies (16, 45, 48).

This study shows that parallel use of electronic and paper-based recording, additional work assignments including participation campaigns, and limited human and financial resources contribute to increased workload and therefore limit eCHIS use. Other studies also showed that maintaining two recording systems has a negative effect on system utilization by increasing workload of CHWs (38, 44, 47, 49, 55, 57). Resource constraints and staff attrition were found to be major barriers to optimal implementation and use of mobile technologies in primary healthcare in line with the findings of this study (36, 38, 40, 46, 48, 50).

Reported benefits of eCHIS over paper-based systems include improved data quality, traceability, retrievability, transparency, and tablet portability. These findings are not peculiar to our study. Various studies identified convenience, ease of access to client data, reduction of errors, improvement in data accuracy, and portability of devices as drivers of technology uptake among primary healthcare workers (43–45, 58–62). This suggests that healthcare professionals value the positive role of technology in healthcare. Hence, removing the barriers and reinforcing the facilitators and motivators will encourage health workers to use the technology to a desirable level.

In this and other studies, health workers reported encouragement from supervisors and colleagues and community recognition and acceptance as motivators to use technologies in their routine work (48, 54, 62).

### 4.1. Strengths and limitations of the study

This study helped provide insights into the study topic as it is primarily based on experience. Several data quality assurance techniques were used to generate quality data from the study. The IDI participants were drawn from six regions allowing for geographical triangulation of the results.

The findings from the study have to be interpreted in light of its limitations. This study did not involve HEWs in the research team and as a result important insights may be lacking. To minimize this problem, eCHIS experts, program implementers and researchers from MOH, universities and digital health partners were involved in the research team. Thus, we recommend that future research involve HEWs in research teams. This study also purposively selected and interviewed the most experienced HEWs. This may result in lack of perspectives from fresh eCHIS users. However, HEWs experience varied from place to place compensating for such limitations.

## 5. Conclusion

Lack of infrastructure and resources; poor quality of training, follow up, and supervision; parallel recording using both the manual and electronic system; and HEW workload are major barriers. Data quality, retrievability, and traceability; tablet portability; cost-effectiveness; encouragement from supervisors; and positive image in the community resulting from HEWs using tablets facilitate the use of eCHIS. Thus, an integrated and coordinated approach that encompasses removing barriers and reinforcing facilitators and motivators is required to promote the use of eCHIS. In accordance with global recommendations by WHO as well as the Community Health Impact Coalition, we call for greater and sustained financial investment to remove the barriers and reinforce the facilitators.

## Data Availability

The raw data supporting the conclusions of this article will be made available by the authors, without undue reservation.
